# Effects of acupuncture for relieving preoperative anxiety in adolescents: A protocol for systematic review and meta-analysis

**DOI:** 10.1097/MD.0000000000028364

**Published:** 2021-12-23

**Authors:** Ziru Yu, Jin Xian, Mi Sun, Wenxiu Zhang, Linwei Li, Xin Zhang, Huijuan Yu

**Affiliations:** a Department of Acupuncture and Moxibustion, Shandong University of Traditional Chinese Medicine, Jinan, Shandong, China; b Affiliated Hospital of Shandong University of Traditional Chinese Medicine, Jinan, Shandong, China.

**Keywords:** acupuncture, adolescents, preoperative anxiety, protocol, systematic review and meta-analysis

## Abstract

**Background:**

Preoperative anxiety is a frequent burden affecting adolescent patients before various surgical procedures. Acupuncture has shown promise for addressing symptoms of preoperative anxiety in adolescents. This study is designed to evaluate the effectiveness of acupuncture for preoperative anxiety in adolescents.

**Methods:**

We will search the relevant randomized controlled trials by the following databases: PubMed, the Cochrane Central Register of Controlled Trials, EMBASE, China National Knowledge Infrastructure, Wan Fang, VIP, China Biomedical Literature Database, and TCM Literature Analysis and Retrieval Database. The process of selecting studies, extracting data and evaluating methodological quality will be conducted by 2 researchers independently. We will use Cochrane risk of bias tool for randomized trials to assess the risk of bias of included studies. Statistical analyses will be performed using R (version3.6.3).

**Ethics and dissemination:**

No patient's privacy are involved in this study, ethical approval will not be required. Our research results are intended to be published through conference reports and peer-reviewed journals.

**INPLASY registration number:**

INPLASY2021110096

## Abstract 2

Supplemental Digital Content is available in the text

## 1 Introduction

Patients usually experience anxiety before various surgical procedures, which is considered as a usual patient response as well as the most frequent burden affecting them.^[1,2]^ There are multiple factors responsible for patient's preoperative anxiety, such as uncertainty, fear, loss of control and decreased self-esteem, when they are facing surgery and hospitalization.^[3]^ Unassessed, untreated, or undertreated preoperative anxiety can cause physiological and pathological effects such as tachycardia, arrhythmias or hypertension, which can lead to undesirable consequences including greater requirement for anesthesia, increased risk of postoperative complications and worse effects of rehabilitation.^[4]^ A recent study has demonstrated that anxiety is most severe in the preoperative waiting area, with only about 7% to 8% patients do not experience preoperative anxiety.^[5]^ Therefore, using a correct approach to avoid possibly preoperative anxiety is very important for the physical and psychological rehabilitation of postoperative patients.

Adolescents are prone to develop anxiety disorders, which is a transitional period from pediatric to adult physiology.^[6]^ Previous studies have shown that adolescents undergoing major surgery, if they have more negatively biased memories for pain, will experience higher postsurgical pain rather than transition to a chronic pain state.^[7]^ Higher levels of postsurgical pain intensity and distress are on account of increased levels of anxiety.^[8]^ Although medications such as anxiolytics and anti-depressants are effective for anxiety, adverse events and side effects must be considered.^[9]^ Thus, the National Institute for Health and Care Excellent in the UK suggested that it is not recommended for routine clinical practice in adolescents.^[10]^

Acupuncture has shown promise in addressing symptoms of preoperative anxiety in adolescents.^[11]^ A series of studies have shown that auricular acupuncture seems to be an effective, safe and easy-to-perform treatment for preoperative anxiety.^[12–14]^ Acupuncture on Yintang point can relieve preoperative anxiety.^[15]^ The mechanism of acupuncture in alleviating anxiety symptoms may be the release of modulators that include β-endorphin, serotonin and substance P with modern medical theory.^[16,17]^ Moreover, acupuncture is a safe and effective method for children and adolescents as a study by Jindal et al found a 1.55 risk of adverse events in 100 treatments of acupuncture.^[18]^ Previous meta-analysis have found that acupuncture therapy plays an active role in preoperative anxiety by decreasing the State Anxiety Subscale and visual analogue scale scores.^[19,20]^ However, a great heterogeneity has been found in those studies because of the age range from children to adults.

Based on the published studies, there is still little high-quality evidence on acupuncture for the treatment of preoperative anxiety in adolescent patients. Therefore, this study aims to evaluate the effectiveness of acupuncture for preoperative anxiety in adolescents.

## 2 Methods

### 2.1 Study registration

This systematic review protocol has been registered in the INPLASY (No. 2021110096). We will follow recommendations outlined in The Cochrane Handbook of Systematic Review of Interventions and the Preferred Reporting Items for Systematic Reviews and Meta-Analysis Protocol statement guidelines. If any adjustments are needed, we will fix and update our protocol to include any changes throughout the study.

### 2.2 Types of studies

Randomized controlled trails will be included, without restrictions on language or publication date, while cohort studies and case reports will be excluded.

### 2.3 Types of participants

Subjects described as adolescent patients undergoing emergency and elective surgery will be included. There will be no restrictions on gender, race and region.

### 2.4 Types of interventions and comparisons

Experimental group: studies using types of acupuncture as the sole intervention, including manual acupuncture, electroacupuncture, auricular acupuncture, transcutaneous electrical acupoint stimulation and acupressure, will be considered.

Control group: trials with comparison interventions, such as sham acupuncture, pharmacological treatment, psychological counseling or no treatment to routine care, will be included.

### 2.5 Types of outcomes

#### 2.5.1 Primary outcomes

Measures of anxiety include the State Anxiety Subscale of the State-Trait Anxiety Inventory.

#### 2.5.2 Secondary outcomes

Visual analogue scale scores, Zung Self-Rating Anxiety Scale or Hamilton Anxiety Scale.

### 2.6 Search methods for identification of studies

All relevant articles will be searched by the following databases from the inception dates to November 1, 2021: PubMed, the Cochrane Central Register of Controlled Trials, EMBASE, China National Knowledge Infrastructure, Wan Fang, VIP, China Biomedical Literature Database and TCM Literature Analysis and Retrieval Database. Taking PubMed as an example, the detailed retrieval strategy is shown in Supplemental Digital Content Appendix S1, http://links.lww.com/MD2/A784.

### 2.7 Data collection

#### 2.7.1 Selection of studies

**Figure 1:**
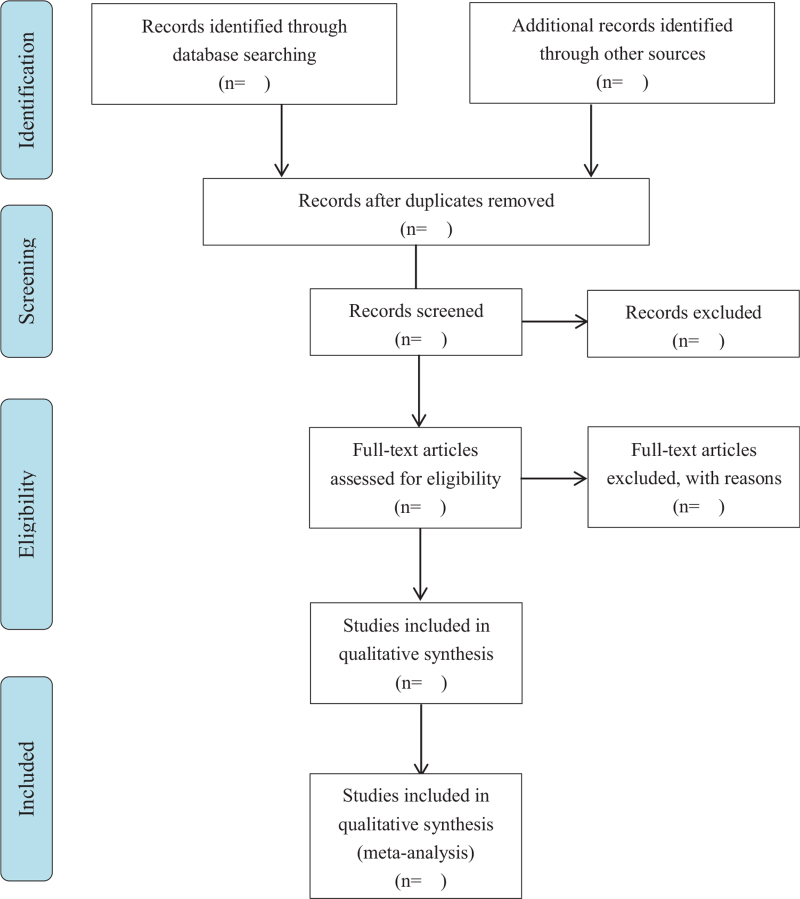
PRISMA flow diagram.

The titles and abstracts, if necessary, full texts of the retrieved studies will be screened by 2 methodological trained reviewers (ZRY and WXZ) in conformity with the established selection criteria independently, with disagreements solves through discussion by a third author (MS). The selection process is presented using a PRISMA flow diagram (Fig. 1).

#### 2.7.2 Data extraction and management

Data from the included studies will be extracted independently by 2 reviewers (ZRY and WXZ), using standardized data extraction form, with the following extracted information from each study: first author, year of publication, language, inclusion and exclusion criteria, sample size, types of interventions of experimental group and control group, outcome measures and adverse events. Any differences in opinion will be resolved by discussion. If failed, a third reviewer (HJY) will be involved for decision.

#### 2.7.3 Quality and bias assessment

Two review authors (LWL and MS) independently will assess the methodological quality and risk of bias of each included trial using the Cochrane Handbook. The assessment details include: random sequence generation, allocation concealment, blinding of participants and personnel, blinding of outcome assessment, incomplete outcome data, selective reporting, and other bias. The bias risk assessment will be divided across 7 dimensions above into 3 criteria: “low risk,” “high risk,” or “unclear risk.” In case of unresolved disagreements, a third reviewer (ZRY) will be available for final resolution.

#### 2.7.4 Dealing with missing data

The corresponding author will be contact for additional data if relevant data is not available. If the missing data cannot be obtained, however, we will exclude the study.

### 2.8 Statistical analysis

#### 2.8.1 Measures of treatment effect

R (version3.6.3) will be used to analyze and synthesize the data. For continuous data, mean differences or standardized mean differences will be calculated. For dichotomous variables, outcomes will be presented as risk ratios with 95% confidence intervals.

#### 2.8.2 Assessment of heterogeneity

Whether to conduct a meta-analysis and which type of analysis model to use (fixed or random effects) will depend on the level of statistical heterogeneity examined by the *P* value and *I*^*2*^ index. If *P* < .05 and *I*^*2*^ > 50%, it will be considered that there is a statistical heterogeneity.

#### 2.8.3 Data synthesis

The fixed effect model will be used if no significant heterogeneity is observed. In contrast, the random effect model will be applied if there is heterogeneity.^[21]^

#### 2.8.4 Subgroup analysis

Based on the results of the data synthesis, subgroup analysis will be conducted to explore possible sources of heterogeneity if sufficient data are available. Subgroup analyses will be conducted according to the types of acupuncture intervention and the different outcomes.

#### 2.8.5 Assessment of reporting bias

The reporting bias will be obtained using R (version3.6.3) by funnel plot and Egger test when more than 10 studies are included.^[22]^

#### 2.8.6 Sensitivity analysis

To confirm the robustness of our results and to determine if the results are affected by the use of different analytical methods, sensitivity analyses will be performed with R (version3.6.3).

#### 2.8.7 Quality of evidence evaluation

Two reviewers (JX and XZ) will assess the evidence quality independently with the Grading of Recommendations Assessment, Development and Evaluation. Considering 5 parameters (publication bias, indirectness, inconsistency, imprecision, and study limitations), we will classify evidence quality into “high,” “moderate,” “low” according to the rating standards.

#### 2.8.8 Ethics and dissemination

No patient's privacy are involved in this study, ethical approval will not be required. Our research results are intended to be published through conference reports and peer-reviewed journals.

## 3 Discussion

More than 90% patients undergoing scheduled for elective surgery developed preoperative anxiety.^[23]^ Acupuncture can provide clinical benefits in the perioperative period, especially for reducing preoperative anxiety, as well as postoperative pain, nausea and vomiting.^[24]^ Previous study indicated that acupuncture might be tolerated and accepted in adolescents suffering from anxiety, with few side effects and adverse events have been reported.^[11]^ Therefore, a systematic review of the effectiveness of acupuncture in the treatment of preoperative anxiety in adolescents is necessary to provide evidence. Our study will provide a reliable and detailed approach for it and the results of this review will benefit clinicians.

## Author contributions

**Conceptualization:** Ziru Yu, Jin Xian, Huijuan Yu.

**Data curation:** Mi Sun, Wenxiu Zhang, Linwei Li.

**Investigation:** Ziru Yu, Mi Sun.

**Methodology:** Jin Xian, Linwei Li.

**Supervision:** Ziru Yu, Wenxiu Zhang.

**Validation:** Jin Xian.

**Visualization:** Ziru Yu, Xin Zhang, Huijuan Yu.

**Writing – original draft:** Ziru Yu.

**Writing – review & editing:** Jin Xian, Huijuan Yu.
